# Theory of Lehmer transform and its applications in identifying the electroencephalographic signature of major depressive disorder

**DOI:** 10.1038/s41598-022-07413-y

**Published:** 2022-03-07

**Authors:** Masoud Ataei, Xiaogang Wang

**Affiliations:** Department of Mathematics and Statistics, York University, Toronto, ON M3J 1P3 Canada

**Keywords:** Neuroscience, Psychology, Mathematics and computing

## Abstract

We propose a novel transformation called Lehmer transform and establish a theoretical framework used to compress and characterize large volumes of highly volatile time series data. The proposed method is a powerful data-driven approach for analyzing extreme events in non-stationary and highly oscillatory stochastic processes like biological signals. The proposed Lehmer transform decomposes the information contained in a function of the data sample into a domain of some statistical moments. The mentioned statistical moments, referred to as suddencies, can be perceived as the moments that generate all possible statistics when used as inputs of the transformation. Besides, the appealing analytical properties of Lehmer transform makes it a natural candidate to take on the role of a statistic-generating function, a notion that we define in this work for the first time. Possible connections of the proposed transformation to the frequency domain will be briefly discussed, while we extensively study various aspects of developing methodologies based on the time-suddency decomposition framework. In particular, we demonstrate several superior features of the Lehmer transform over the traditional time-frequency methods such as Fourier and Wavelet transforms by analyzing the challenging electroencephalogram signals of the patients suffering from the major depressive disorder. It is shown that our proposed transformation can successfully lead to more robust and accurate classifiers developed for discerning patients from healthy controls.

## Introduction

Analysis of the non-stationary spatiotemporal data is often a daunting task as it involves the mediation of the concepts, theories, and techniques often developed in several seemingly diverse areas of mathematical, statistical, engineering and computational sciences. Most notably, the non-stationarity of time series can be shown to have a subtle connection to the non-autonomous behavior of dynamical systems in which the oscillatory law governing the manifestation of a specific phenomenon itself undergoes various temporal changes. As a result, acquiring a relatively thorough understanding of any phenomenon that gives rise to the realization of highly oscillatory and complex time series data can only be accomplished simultaneously utilizing the instruments developed in statistical and dynamical systems theories.

Even though the concurrent use of the classical statistics framework alongside the dynamical systems theory can potentially encapsulate many valuable information about the observed time series data, little does it reveal the temporal nature of the laws governing widespread non-stationary processes. For instance, the non-autonomous behavior of the oscillatory systems is typically attributed to the influence of some external fields; in this respect, the dynamical systems theory could be a potential aid for analysis insofar as the external modulation source is known or strictly assumed to be deterministic, periodic, or stochastic. In a situation where the governing law of the external modulations itself may exhibit some sort of time-dependency, such approaches for studying the phenomena usually become less effective.

This work presents a data-driven model based on a new class of transformations to reliably infer the underlying laws of the non-stationary phenomena based on the time series data observed. The proposed mathematical model is shown to be a framework *par excellence* to exploit possible sources contributing to the occurrence of the extreme events in non-stationary stochastic processes and non-autonomous dynamics. To this end, we introduce, for the first time, the notions of the Lehmer transform and the so-called domain of suddency moments. Briefly stated, Lehmer transform decomposes the information contained in a function of the data sample into their constituting statistical moments. In other words, the Lehmer transformation can be perceived as domain representation on a space of certain statistical moments, which we refer to them as the suddency moments (or *s*-moments in short).

The domain of *s*-moments shares a number of similarities with the widely used frequency domain. However, unlike the frequency domain analysis whose focal point is the intrinsic periodicity of all phenomena observed in the nature, our proposed analytical framework frees the statistical moments domain from adhering to any restrictive assumptions such as the one mentioned above. We find it meritorious to mention that, the proposed Lehmer transform and its statistical moment domain can provide a set of complementary instruments for the traditional frequency-based frameworks, if not replace them entirely. In turn, this further enables one to construct parametric families of non-parametric statistical models, potentially bridging the existing gap between parametric and non-parametric areas of statistics.

Our proposed transform, named after the renowned number theorist Derrick H. Lehmer, has its mathematical roots in generalized means family. In particular, there exists a member of the mentioned family of functions known as the Lehmer mean, which has primarily been studied in specific branches of analytic number theory, to the best of our knowledge. Nevertheless, the appealing properties of this function have not received much attention from statistics, machine learning and data science communities in the past, leaving its wide range of potential applications largely unexploited in handling complex non-stationary time series such as biological signals.

Most existing works employing the Lehmer mean function for the purpose of data analysis, rely mainly on its special cases. For instance, Terziyan^21^ formulated a distance metric using the harmonic and contra-harmonic means and evaluated its effectiveness in geographic information systems. In a similar manner, Somasundaram et al.^20^ studied the disconnected graphs and their possible labeling schemes and Sluciak^19^ developed state-dependent consensus algorithms by resorting to applications of some special cases of the Lehmer mean function. Besides, Gomes^11^ successfully constructed a family of high-performance value at risk estimators through the use of this function and proved asymptotic normality of the constructed estimators.

On the other hand, being a generalization of the power mean function and having connections to other important classes of means, more attention has recently been geared towards the Lehmer mean function and its characterization. For instance, the function’s elementary properties like homogeneity, monotonicity and differentiability have been discussed in^1,3^, whereas its more advanced properties like Schur-convexity, Schur harmonic convexity and Schur power convexity have been the focus of more recent studies, e.g., see^8–10,23,24^ and references therein. Also, the inflection points of the function have been studied in^18^, and the results concerning its possible connections to Gini and Toader means have been provided in^5–7,12,22,25^.

In this article, we formally define the Lehmer transform and the domain of *s*-moments. Thereafter, we use the developed framework to study a benchmark electroencephalography (EEG) dataset of patients suffering from major depressive disorder (MDD), whose computational results are further provided and discussed in detail. Before that, however, we find it of great importance to clarify our motivations for introducing and laying out the theoretical foundations of this new transformation within the context of brain wave analysis and, in particular, the EEG analysis of MDD dataset.

## Motivation

The proposed Lehmer transform is intrinsically a powerful method to summarize the information contained in any given set of data, whether sequential data like time series or unordered ones. More explicitly, this transform computes the ratio of two moments whose numeratorial and denumeratorial power difference is exactly one. Even though it might not be entirely clear at first glance, this mechanism leads to unraveling significant amount of salient information contained in the data that other transforms cannot capture.

On the one hand, from the statistical physics point of view, one would look at a given energy level through the use of various powers for the *s*-moments. On the other hand, from the classical statistics perspective, the Lehmer transform can be perceived as a statistic-generating function, i.e., one can generate all possible statistics such as sample mean, maximum, minimum, etc., by applying the Lehmer transform to specific values of the *s*-moments. Accordingly, all complex non-stationary signals can be formulated under one coherent and unified framework capable of concentrating on patterns that are difficult to capture otherwise by Wavelets or Fourier transforms.

More specifically, the main challenges and limitations confronted by analysts equipped with the frequency domain tools for analysis of EEG signals can be surmounted to a great extent by resorting to applications of our proposed framework:

1. **Filtering the noise:**
  The raw EEG signals contain various artifacts arising from eye movements (horizontal and vertical), eye blinks as well as muscular and heart activities which require applying a set of suitable filtering techniques in the prepossessing step. However, the majority of the filtering techniques, in one way or another, require the data to satisfy certain number of conditions on stationarity. The EEG signals rarely meet such requirements in reality, rendering inefficacy of many filtering techniques used for noise removal.
  The most common procedures used for removing noise from EEG signals are semi-automatic. That is, the subjective opinion of a well-trained operator plays a key role in selecting the types of artifacts to be corrected. As a result, the noise filtering techniques applied to a certain dataset become to some extent customized based on the choices made by the operator during artifact removal stage. Thus, the frameworks and methodologies borrowed from any other study should be incorporated with great caution on side of fully understanding the steps conducted by previous scholars. What we pointed out above can arguably be considered among the most substantial factors leading to lack of portability of the vast majority of the methods available from the literature, partially delineating the reasons as to why various inconsistencies are observed across different lines of study on psychiatric disorders and neuroscience areas.
2. **Lack of interpretability:**
  Most of existing models which rely on frequency-domain properties, usually assemble a complex of advanced-level mathematical ideas and tools to solve the problems. However, this cannot be achieved without compromising the interpretability of the model and its subsequent results, whose impacts become even more pronounced for those researchers not already mastered the countless venues of Fourier and Wavelets analyses, who would face various impediments upon their attempt to build up an intuititive know-how of such approaches. After all, it is well-known that a thorough and insightful understanding of the brain and its underlying mechanisms is more likely to be obtained through devising interpretable mathematical models instead of convoluted ones.
3. **Existence of various dependence relations:**
  In studying the brain by analyzing its EEG signals, it is essential to consider the fact that various clusters of neurons are spatially related to one another, especially the association and dependence being stronger in the neighboring clusters. However, the EEG signals quantify the local activities of the brain. As a result, the spatial relations among various clusters of neurons which reside on a three-dimensional space get mapped to the two-dimensional surface of the scalp through EEG recordings, rendering the loss of all spatial information previously-existing among the clusters and their connecting electrodes.
  An important implication here is that the inter-cluster dependence relations which are apparent in the recorded EEG signals, do not necessarily reflect the true spatial relations among different regions of the brain. Omitting investigation of existing dependence relationships can make the outcomes of the analysis rather unreliable. The difficulty for exploiting those spatial relations become even more concerning when the non-stationary and temporal nature of the data are further taken into account.
4. **Dealing with big volumes of data:**
  It is no secret that the data recorded by EEG electrodes can easily exceed terabytes of volume, especially when the brain waves are recorded for extensive periods of time in order to detect an anomalous behavior as a symptom of severe disease. Hence, incorporation of big data technologies such as cloud computing is inevitable. The main issue here is not lack of accessibility to adequate amount of memory for storing the data, rather it pertains to the fact that analysis of big volumes using current frequency-based methodologies are simply not feasible due to the fact that almost all those methods rely on subroutines for traversing over large number of variable-size frequency windows and their countless combinations during fine-tuning the parameters step. In other words, it is the lack of any just-in-time framework for analysis of EEG signals that demands for storage of big volumes of data.
5. **Non-applicability of many theoretical frameworks:**
  Presence of large volumes of data naturally calls for the use of statistical theories in order to summarize the EEG data and reduce their dimensions. However, it is not straightforward at all to extend the results of the standard statistical theories to the situations where random variables involved in the system demonstrate strong evidence of dependence and they almost never are identically distributed. Even resorting to the asymptotic theories of statistics which allow violation of several restrictive constrains often renders impractical due to the extremely small sample sizes available, especially when the cases related to some rare diseases are studied whose number of participants is limited.
6. **Handling the missing data:**
  The EEG studies often entail several experiments, but it is very common to have a dataset in which the recordings pertaining to some experiment(s) for a particular subject are entirely missing, i.e., the recordings related to all (or some) of the electrodes are missing for the entire period of the experiment(s). However, it is not always possible to remove that particular subject from the list of participants in order to handle the missing data, e.g., the number of participants might not be large enough for this purpose. More importantly, the missing data are often associated with some of the key participants of the study, whose removal can induce substantial bias in the final outcome of analysis. Various types of censoring and truncation are among the potential reasons behind missing data.
  Besides, the nature of censorship or truncation can be of such a type that partial recordings are available for that particular person under the experimentation(s). In those cases, one cannot easily omit the participant from the dataset as those data, even not being complete, convey important information about the subject and can contribute to discovering various subtleties not apparent about the psychiatric disorder under study. The set of techniques prescribed by the survival analysis can prove useful in those situations, however most of the existing models, especially the ones developed in frequency domain, do not extend easily to accommodate applications of the survival analysis techniques.

Now, we briefly list out all the advantages that our proposed framework based on the Lehmer transform possesses as follows:

1. Performing the preprocessing step for noise filtering is entirely eliminated in the proposed framework due to the fact that *s*-moments capture all the noise polluting the EEG signal, regardless of the source which has generated the noisy data, further enabling one to bypass the filtering step of EEG signal analysis.
2. The model is easily interpretable by those lacking sufficient knowledge on technical details related to the areas of mathematics and physics since the proposed framework enables one to deal with the familiar and tangible notion of statistics such as the sample mean. Furthermore, the Lehmer transform directly works with the actual measurements recorded on the EEG electrodes (measured in mV) without requiring application of any transformation. More importantly, this facilitating feature of the model lends itself to our proposed action potential function that lets one study the brain activities on a true scale of the action potentials of neurons, which is the most intuitive approach and leads to many insightful observations and discoveries on brain functionality.
3. Since our model utilizes all possible summary statistics to describe the data, there is no need to store large volumes of data anymore. In other words, all the computations can be carried out online with almost negligible space and time complexities, making it one of the most suitable frameworks to utilize when the EEG experiments are designed to last for relatively longer periods of time.
4. Spatiotemporal-preserving structure of our model in the domain of *s*-moments makes it practical to apply a vast number of machine learning techniques for both supervised and unsupervised learning purposes. The reason is that many machine learning algorithms require large number of sample points with dimensions on feature space of the problem being as low as possible to guarantee their success, which can easily be accomplished through the use of our proposed statistical moments.
5. Being itself a statistical model in nature, our proposed framework allows incorporation of the tools developed in various areas of statistical sciences such as survival analysis in order to deal with the censored and missing data.
6. A great advantage of the proposed model is that it enables one to detect existence of possible sub-types, either by visual inspection or the use of algorithmic approaches. Our developed action potential probability distribution function will be the main gauge to visualize the multi-dimensional EEG data, which can prove extremely beneficial at both the preliminary stages of the study and during the algorithm design process.

## Lehmer transform

Let ${\mathbb {R}}_{>0}$ and $\overline{{\mathbb {R}}}$ denote the sets of strictly positive and extended real numbers, respectively. Given a signal $\mathbf {x}=\{x_i \in {\mathbb {R}}_{>0}, i=1,\dots ,n\}$, the Lehmer transform ${\mathscr {L}}:\overline{{\mathbb {R}}}\rightarrow {\mathbb {R}}_{>0}$ is a mapping defined by

$$\begin{aligned} {\mathscr {L}}({s}) = {\left\{ \begin{array}{ll} \max \limits _{i=1,\dots ,n}\left\{ x_i\right\} , &{} \quad \text{ if } \ s = \infty , \\ \dfrac{\sum \limits _{i=1}^n x_i^s}{\sum \limits _{i=1}^n x_i^{s-1}}, &{} \quad \text{ if } \ s \in (-\infty ,\infty ), \\ \min \limits _{i=1,\dots ,n}\left\{ x_i\right\} , &{} \quad \text{ if } \ s = -\infty . \\ \end{array}\right. } \end{aligned}$$

According to this definition, the Lehmer transform ${\mathscr {L}}({s})$ maps every point $s \in \overline{{\mathbb {R}}}$ into some statistic contained within the range $[ \min \{\mathbf {x}\} , \max \{\mathbf {x}\} ]$, where we refer to the point *s* as a suddency moment of the given signal $\mathbf {x}$. Table 1 reports some of the widely-encountered suddency moments in data analysis. The proposed transform is differentiable in *P*-probability and its *n*th order derivative could be proven using the mathematical induction to be as follows:

$$\begin{aligned} \dfrac{\partial ^n}{\partial s^n} {\mathscr {L}}({s}) = {\mathscr {L}}({s}) \left[ \sum _{k=0}^{n} \dfrac{1}{k!} \left( \sum _{j=0}^{k} \left( -1\right) ^j {k \atopwithdelims ()j} \, \Lambda ^{j} \, \dfrac{\partial ^n}{\partial s^n} \Lambda ^{k-j} \right) \right] , \end{aligned}$$

where

$$\begin{aligned} \Lambda := \log {\mathscr {L}(s)}. \end{aligned}$$

In particular, the first derivative of the Lehmer transform is derived as

$$\begin{aligned} \dfrac{\partial }{\partial s} {\mathscr {L}}({s}) =\, {\mathscr {L}}({s}) \left[ \dfrac{\sum \limits _{i=1}^n x_i^s \log x_i}{\sum \limits _{i=1}^n x_i^s} - \dfrac{\sum \limits _{i=1}^n x_i^{s-1} \log x_i}{\sum \limits _{i=1}^n x_i^{s-1}} \right] =\, \sum _{i=1}^{n} \sum _{k=i+1}^{n} \left( x_i - x_k \right) \left( \log \frac{x_i}{x_k} \right) \left( x_i x_k \right) ^{s-1}. \end{aligned}$$

**Table 1:** Some special cases of suddency moments.

Suddency domain	Sample domain
$-\infty$	Minimum ($n\ge 1$)
0	Harmonic mean ($n\ge 1$)
1/2	Geometric mean ($n = 2$)
1	Arithmetic mean ($n\ge 1$)
2	Contra-harmonic mean ($n\ge 1$)
$\infty$	Maximum ($n\ge 1$)

Furthermore, for any statistic $t\in [ \min \{\mathbf {x}\} , \max \{\mathbf {x}\} ]$, the inverse image $\mathscr {L}^{-1} : {\mathbb {R}}_{>0} \rightarrow \overline{{\mathbb {R}}}$ provides a set that contains the associated suddency moment(s) of the signal such that

$$\begin{aligned} {\mathscr {L}}^{-1}({t}) = \left\{ s \in \overline{{\mathbb {R}}} : {\mathscr {L}}({s}) = t \right\} . \end{aligned}$$

Subsequently, given that the signal $\mathbf {x}$ contains at least two unique elements, the Lehmer transform ${\mathscr {L}}({s})$ will be a monotonously increasing function, and its inverse transform can be obtained by resorting to the following theorem:

### Theorem 1

Given the signal $\mathbf {x}$ satisfying the condition $x_i \ne x_j$ for some $i,j\in \{1,\dots ,n\}$ such that $i\ne j$, the inverse Lehmer transform is defined as

$$\begin{aligned} {\mathscr {L}}^{-1}({t}) = {\left\{ \begin{array}{ll} \infty , &{} \text{ if } \ t = \max \limits _{i=1,\dots ,n}\left\{ x_i\right\} , \\ g(t), &{} \text{ if } \ t\in \left( \min \limits _{i=1,\dots ,n}\left\{ x_i\right\} , \max \limits _{i=1,\dots ,n}\left\{ x_i\right\} \right) , \\ -\infty , &{} \text{ if } \ t = \min \limits _{i=1,\dots ,n}\left\{ x_i\right\} , \\ \end{array}\right. } \end{aligned}$$

where

$$\begin{aligned} g(t) = s_0 + \sum \limits _{k=1}^{\infty } \dfrac{\left( t-{\mathscr {L}}({s_0})\right) ^k }{k!} \left\{ \lim \limits _{s\rightarrow s_0} \left[ \dfrac{\partial ^{k-1}}{\partial s^{k-1}} \left( \dfrac{s-s_0}{{\mathscr {L}}({s}) - {\mathscr {L}}({s_0})} \right) ^k \right] \right\} , \end{aligned}$$

for some $$s_0\in {\mathbb {R}}$$.

### Proof

Under the compatibility condition imposed on the given signal, the monotonicity property of ${\mathscr {L}}({s})$ implies that the function is injective, which in turn guarantees the existence of some inverse function denoted by ${\mathscr {L}}^{-1}({t})$. By further noting that ${\mathscr {L}}({s})$ is analytic for every point $s\in {\mathbb {R}}$, the Lagrange-Bürmann formula^4,15^ can be utilized to express the interior points of the inverse function as a Taylor expansion *g*(*t*) about some given point $s_0\in {\mathbb {R}}$, hence yielding the presented result. $\square$

The following example employs the inversion theorem mentioned above and illustrates an application of the inverse Lehmer transform as a non-linear filter for detecting anomalous events.

### Example 1

**Figure 1:**
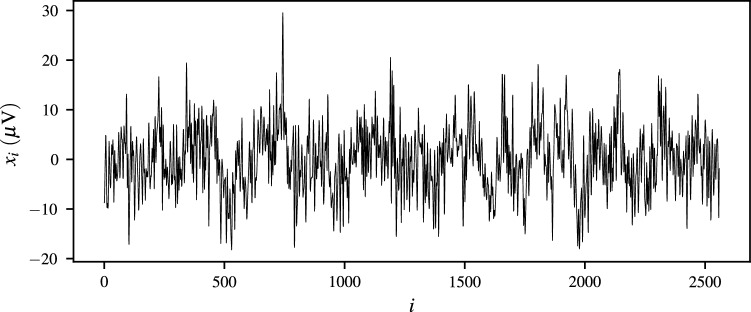
Plot of raw membrane potentials against time.

**Figure 2:**
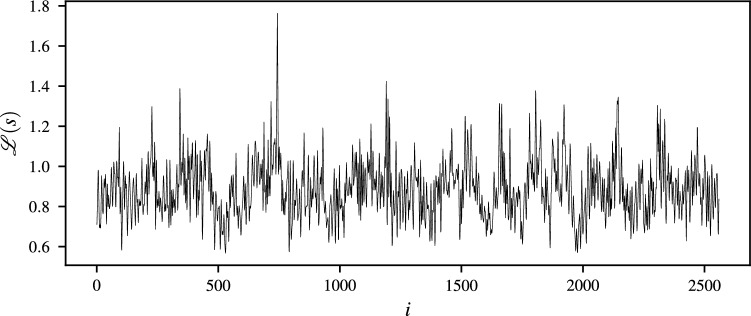
Plot of Lehmer transform against time.

**Figure 3:**
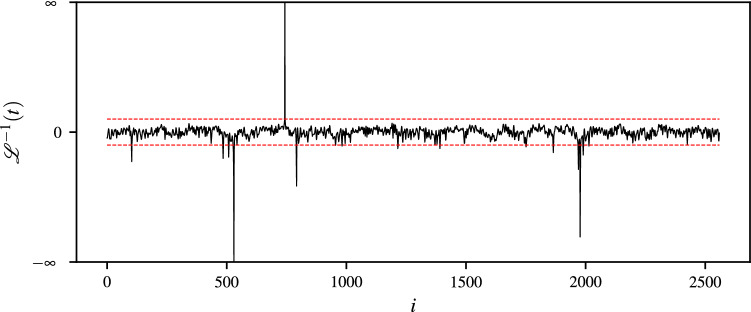
Plot of inverse Lehmer transform against time.

Let us assume that a time-indexed sample $\mathbf {x}=[x_1,x_2,\dots ,x_n]^\intercal$ is given whose elements correspond to the EEG measurements recorded using ${256}\,\mathrm{Hz}$ sampling frequency for a time period of 10 seconds. Figure 1 depicts the plot for membrane potentials measured at every time index $i=1,2,\dots ,n$. Furthermore, Figs. 2 and 3 exhibit the plots corresponding to Lehmer transform and its inverse function, respectively. As revealed by Fig. 2, ${\mathscr {L}}({s})$ successfully perpetuates structure of the raw EEG signal. Besides, Fig. 3 shows that the majority of computed $s-$moments lie in a range $s\in \left( -20,20\right)$, where the thresholds are indicated using dashed lines. Examining the EEG signal on a statistical moment domain, may then help one to detect the anomalous points and identify the time-dependent behavior of their underlying events.

## Action potential distributions

In this section, we show that the Lehmer transform has a deep connection with potential functions. It is used to construct a general class of action potential functions that are suitable to characterize intrinsic patterns embedded in a non-stationary time series such as EEG signals.

**Figure 4:**
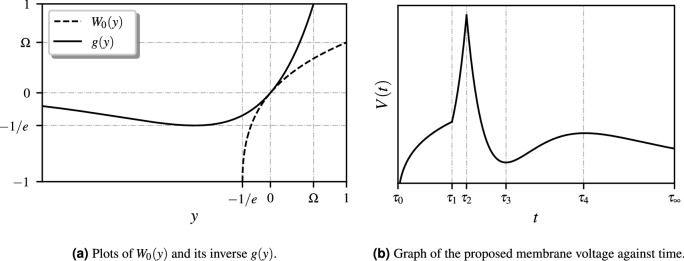
Schematics of $W_0(y)$, *g*(*y*) and *V*(*t*).

Let us recall the principal branch of the Lambert-*W* function denoted by $W_0(z)$, which is defined for every real $z\in \left( -e^{-1},\infty \right)$ as the inverse function of $g(z) = z e^z$, whose schematics are depicted in Fig. 4a. Here, $\mathrm {\Omega }$ denotes the Omega constant defined by $\mathrm {\Omega }=W_0(1) \approx 0.5671.$ The membrane voltage function (see Fig. 4b) may then be expressed as some transformation of the following functional (for a constant *c*):

$$\begin{aligned} V(t;c)= {\left\{ \begin{array}{ll} W_0(t-\dfrac{1}{e})- \dfrac{1}{ce} , &{} \quad \text {if } t\in \left( 0,\dfrac{1}{e} \right) , \\ t^2 e^t - \dfrac{1}{e} , &{} \quad \text {if } t\in \left( \dfrac{1}{e},\dfrac{e+1}{e} \right) , \\ t^2 e^{-t} - \dfrac{1}{e} , &{} \quad \text {if } t\in \left( \dfrac{e+1}{e}, \infty \right) . \end{array}\right. } \end{aligned}$$

Inspired by the appealing properties of the membrane voltage function defined above, we propose a distribution function to account for the probabilistic phenomena that underlie the complex dynamics of the action potentials.

### Theorem 2

Let us assume that a signal is given which has been transformed such that it would satisfy the following conditions:

$$\begin{aligned} {\mathscr {L}}(\infty ) = \exp \{\frac{W_0(\alpha \beta )}{\alpha \beta }\} \quad \text {and} \quad {\mathscr {L}}(-\infty ) = \exp \{W_0(0)\}. \end{aligned}$$

Then, a real-valued random variable *S* is said to follow an action potential distribution if its probability density function is given by

$$\begin{aligned} f_S(s) = \frac{C}{\alpha } \left( 1+\alpha \beta {\mathscr {L}}({s}) \right) e^{\beta {\mathscr {L}}({s})} ({\mathscr {L}}({s}))^{\frac{1}{\alpha }-1} \mathscr {L}^{\prime }(s), \end{aligned}$$

where $\alpha \in \left( 0,1\right]$, $\beta \in {\mathbb {R}}_{>0}$ and the normalization constant *C* is obtained using the following relation

$$\begin{aligned} C = \left( \exp \left\{ \frac{W_0(\alpha \beta )}{\alpha ^2 \beta } + \beta \exp \{\frac{W_0(\alpha \beta )}{\alpha \beta }\} \right\} - \exp \left\{ \beta \right\} \right) ^{-1}. \end{aligned}$$

### Proof

Let us consider the following function

$$\begin{aligned} F(s) = A + B ({\mathscr {L}}({s}))^{\frac{1}{\alpha }} e^{\beta {\mathscr {L}}({s})}, \end{aligned}$$

where

$$\begin{aligned} A = \dfrac{\exp \left\{ \beta \right\} }{\exp \left\{ \dfrac{W_0(\alpha \beta )}{\alpha ^2 \beta } + \beta \exp \{\dfrac{W_0(\alpha \beta )}{\alpha \beta }\} \right\} - \exp \left\{ \beta \right\} } \end{aligned}$$

and

$$\begin{aligned} B = \dfrac{1}{\exp \left\{ \dfrac{W_0(\alpha \beta )}{\alpha ^2 \beta } + \beta \exp \{\dfrac{W_0(\alpha \beta )}{\alpha \beta }\} \right\} - \exp \left\{ \beta \right\} }. \end{aligned}$$

Then, it is straightforward to show that *F*(*s*) is right-continuous and nondecreasing in the domain of suddency moments, and further it satisfies the following properties

$$\begin{aligned} \lim _{s\downarrow -\infty }F(s) = 0 \quad \text {and} \quad \lim _{s\uparrow \infty }F(s) = 1. \end{aligned}$$

Thus, *F*(*s*) is a distribution function. As a result, we may resort to the Caratheodory extension theorem which asserts that there exists a unique Lebesgue-Stieltjes measure associated with the distribution function *F*(*s*). In turn, this enables us to define a real-valued random variable *S* on a support $s\in (-\infty ,\infty )$, whose cumulative probability distribution function, i.e., $F_S(s)$, coincides with *F*(*s*). The probability distribution function presented in the theorem is then derived by differentiating the $F_S(s)$. $\square$

**Figure 5:**
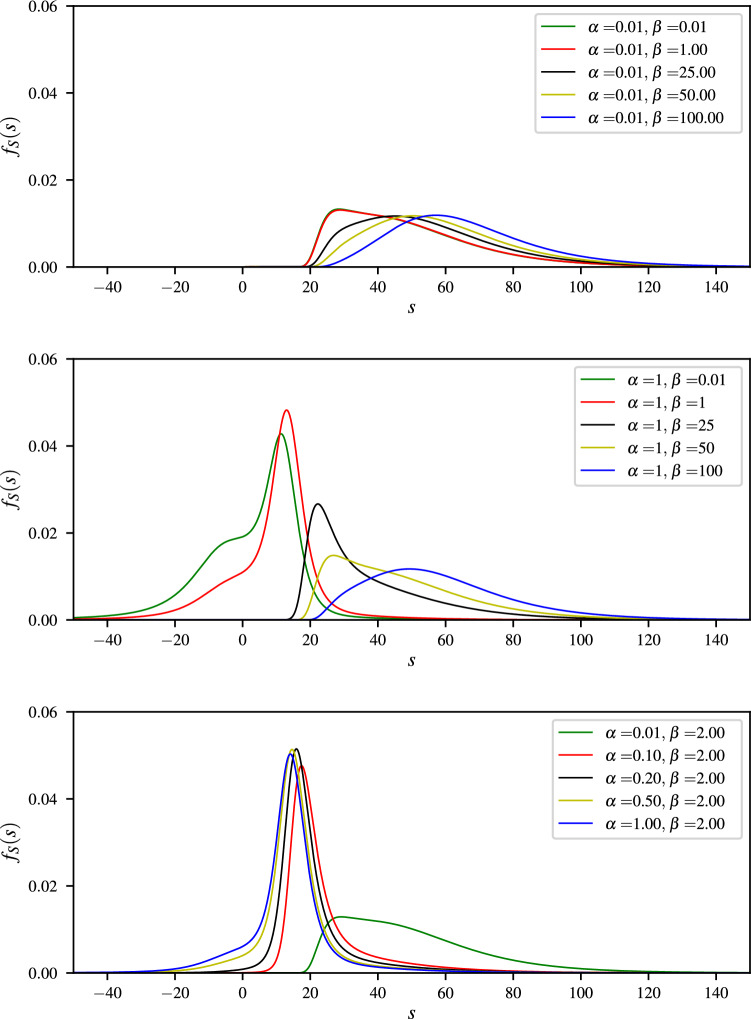
Plots of action potential distributions for some EEG signal with varying the parameters $\alpha$ and $\beta$.

The action potential distribution function is a general framework to analyze complex non-stationary time series such as brain waves, e.g., see Fig. 5 which depicts plots of action potential distribution function for an EEG signal with varying the parameters $\alpha$ and $\beta$. An important class of action potentials is referred to as the dendritic spikes, which plays a significant role in neuronal communication, memory and learning.

Theoretically speaking, the dendritic spike dynamics would involve a number of different phase transitions, typically starting out from an initialization phase upon allying a stimulus. The dynamical system would then enter a rising phase if the membrane voltage rises above some threshold value, which is then followed by a phase transition into a falling phase. The final transition would occur at a reestablishment phase through which the membrane potential would eventually reside at its resting state.

In reality, however, a large body of evidence in neurophysiology suggests that the variations in electro-physiological measurements may distort the actual action potentials, yielding a substantially different phase transition scheme as compared to the theoretical one mentioned above. That is, different types of undershoot and overshoot may be encountered in dendritic spikes dynamics. The key element which we incorporate in our analysis of dendritic spikes, pertains to the point that membrane voltages could effectively be modelled using the Lambert-*W* function and its variants.

## Major depressive disorder

The data set we study in this work is comprised of EEG recordings of 34 patients suffering from MDD. This is one of the benchmark data sets to test classification algorithms designed for EEG recordings and extract knowledge on the underlying mechanisms and functionality of different areas of the brain in MDD patients. In this research, a sample of 34 MDD outpatients with 17 males and 17 females was recruited according to the experiment design at the Hospital University Sains Malaysia (HUSM), Kelantan, Malaysia. During each visit to the clinic, the MDD patients were assessed by experienced clinicians. Detailed information can be found in^17^.

Our primary goal is to investigate whether the patterns existing in the EEG recordings of the subjects can be used for discerning the MDD patients from healthy controls. To this end, EEG recordings of 30 control subjects are further taken into consideration as the control group of this study. Each subject undergoes 3 different types of experiments while his/her EEG signals are recorded. More precisely, the experiments are conducted in 3 different setups: eyes closed (EC), eyes open (EO), and while the subject performs a specific task (TASK). The duration for each of the experiments EC and EO is 5 minutes which comprises an average of 77000 signals per each channel (there exist 19 channel per each experiment). The experiment TASK, however, lasts for longer duration 10 minutes and its average number of signals sampled with frequency 256Hz is roughly 155000 on each channel; see^17^ for more details on the conducted experiments.

One of the challenges faced when studying the present problem pertains to the fact that a considerable number of data are missing from the provided dataset. Summary of the missing data for each of the experiments within control and MDD groups are presented in Table (2). Furthermore, let us use matrices to embed the experiment data by assigning their columns to different channels and populating their rows based on the EEG samples observed in the time domain (measured in $\mathrm {mV}$). In other words, the EEG recordings of the *n*th subject, $n=1,2,\dots ,N$, within the *m*th experiment, $m=1,2,\dots ,M$, is denoted using the following matrix

$$\begin{aligned} \mathbf {X}^{(n,m)} = \left[ x_{ij} \right] _{I\times J} \end{aligned}$$

where $N=64$ and $M=3$ are total number of the subjects and experiments, respectively. Also, $J=19$ denotes total number of the active channels within each experiment where the recordings on each of these channels contain *I* samples points.

**Table 2:** Summary of missing data for MDD dataset.

Experiment	EC	EO	TASK	Total
Control	6.7%	3.3%	6.7%	5.5%
MDD	11.8%	5.9%	2.9%	6.9%
Total	9.4%	5.0%	5.0%	6.3%

**Figure 6:**
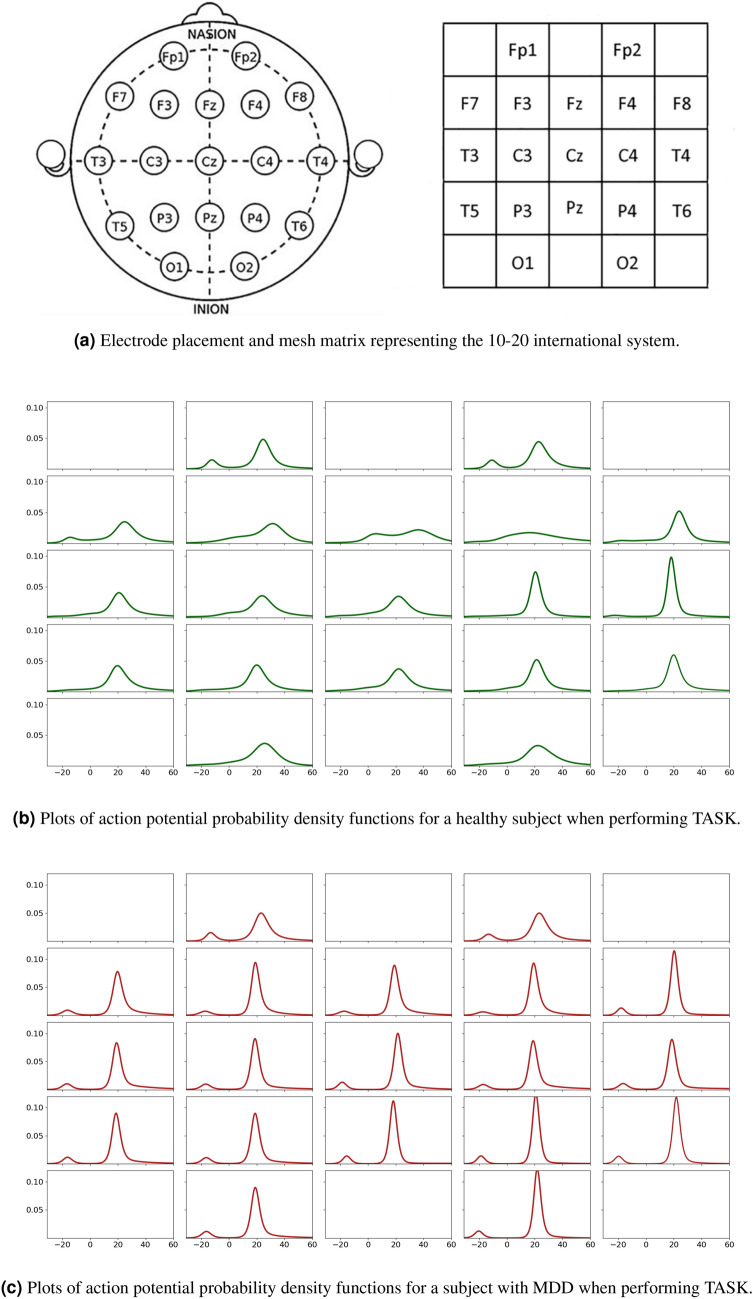
Schematics of electrode placement and action potential distributions for control and MDD subjects.

The key point in our developed methodology is to assign random variables to each channel using the action potential distribution. In this way, one would take an implicit approach in studying the set of random vectors which have spatiotemporal correlations and autocorrelations as well as various other types of dependence. That is, for each signal contained within the dataset corresponding to a single column of some matrix $\mathbf {X}^{(n,m)}$ for the subject *n* and experiment *m*, parameters of the action potential distribution are estimated using the maximum likelihood method. Figure 6 depicts plots of 19 action potential probability density functions for EEG signals of a single healthy subject when performing the TASK, which are compared to 19 probability density function of a subject selected from the MDD group.

**Figure 7:**
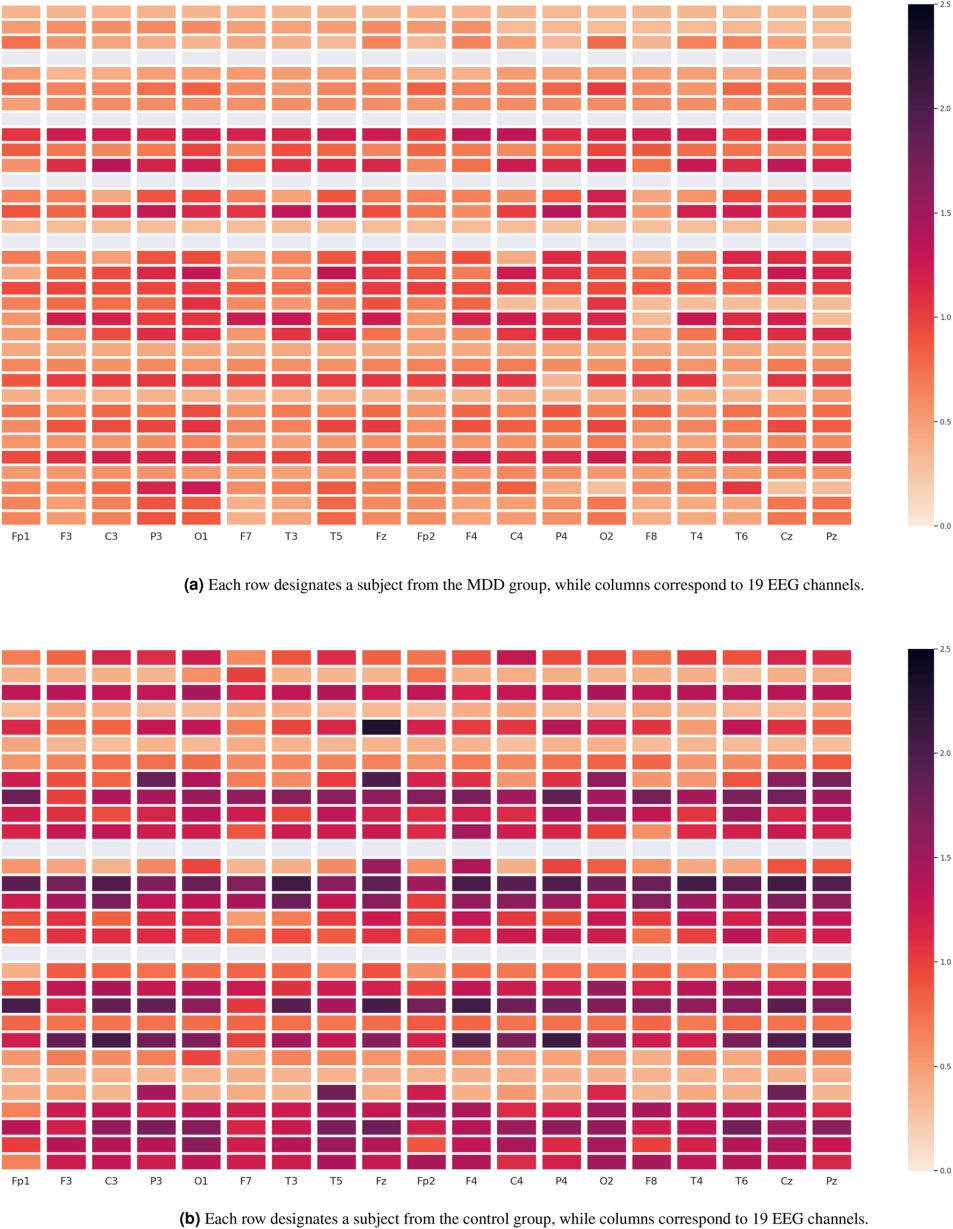
Heat maps of the differential entropy of action potential distributions for experiments with EC.

**Figure 8:**
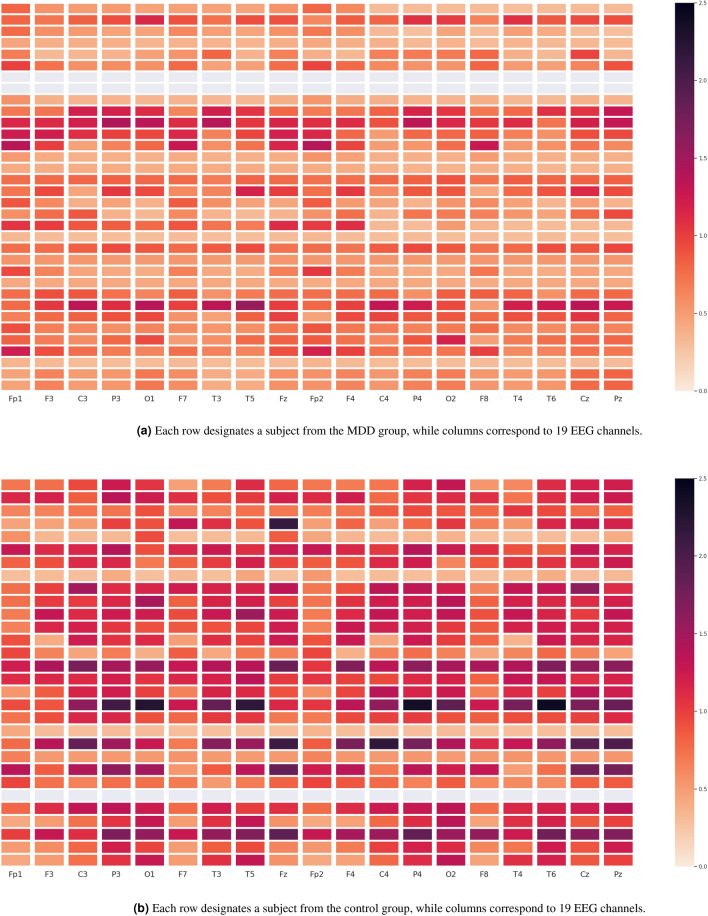
Heat maps of the differential entropy of action potential distributions for experiments with EO.

**Figure 9:**
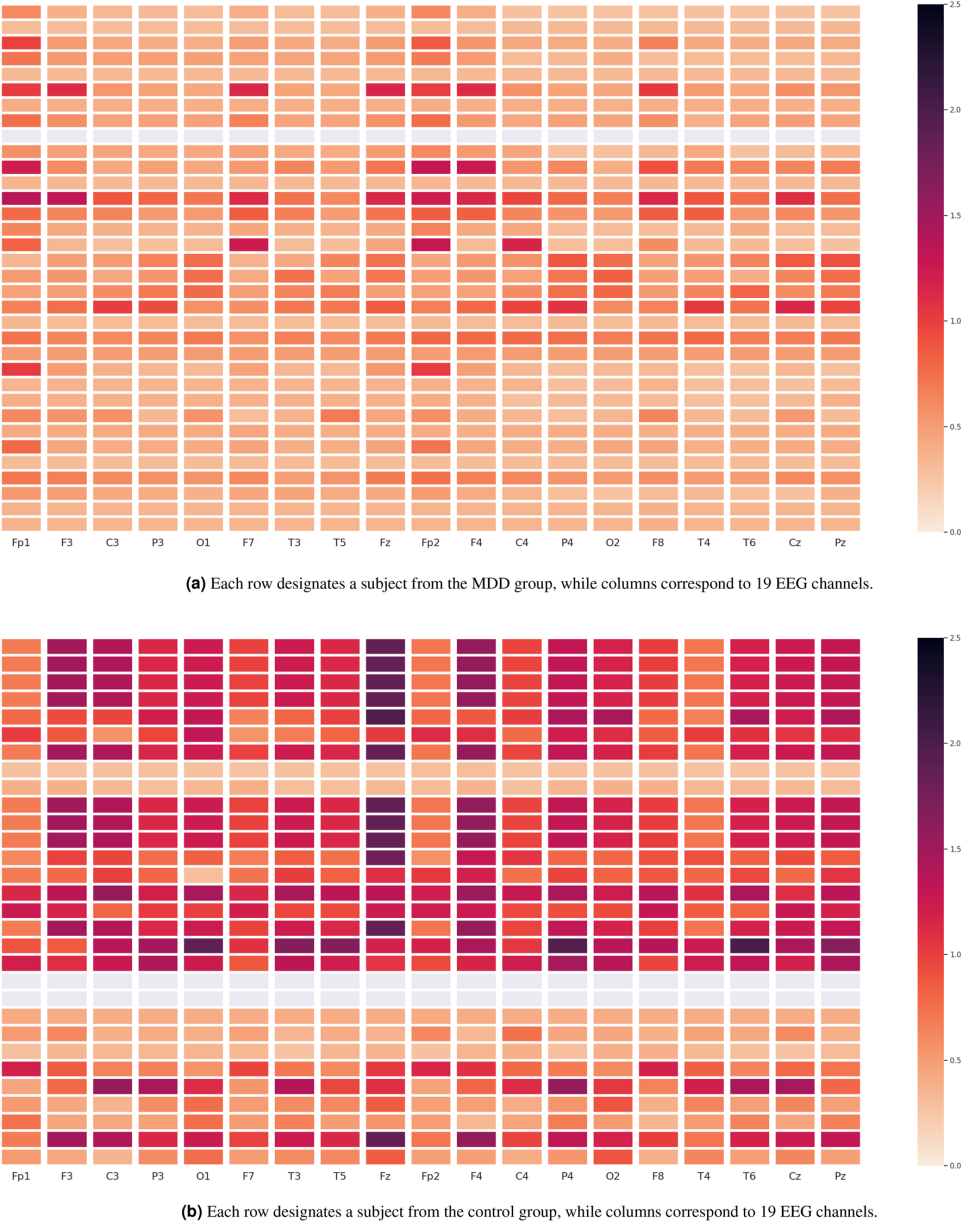
Heat maps of the differential entropy of action potential distributions when performing TASK.

Furthermore, Figs. 7, 8, 9 depict the heat maps constructed based on differential entropy values computed for the action potential distribution functions of all available EEG signals. It is observed that the overall brain activation of MDD patients rests at its lowest possible state across all three experiments, whereas their brain becomes relatively more active overall when they undergo the EC and EO experiments. It is also interesting to note the consistently low activation of the temporal regions of the brain, especially the right ones, across all experiments.

Next, the following quantities are computed for each available signal using its underlying action potential distribution function. That is, for the EEG signal recorded on the *j*th channel of experiment *m* associated to subject *n*, we compute:

$$\begin{aligned} \int \limits _{-\infty }^{{\mathfrak {m}}_1} f(s) \log f(s) ds, \quad \int \limits _{{\mathfrak {m}}_1}^{{\mathfrak {m}}_2} f(s) \log f(s) ds, \quad \int \limits _{{\mathfrak {m}}_2}^{\infty } f(s) \log f(s) ds,\quad F({\mathfrak {m}}_1) \quad \text {and} \quad 1 - F({\mathfrak {m}}_2), \end{aligned}$$

where *f* and *F* represent $f^{(m,n)}_j$ and $F^{(m,n)}_j$, respectively. Furthermore, we define ${\mathfrak {m}}_1$ and ${\mathfrak {m}}_2$ as follows:

$$\begin{aligned} {\mathfrak {m}}_1 = \underset{{s \le 1}}{\arg \max } f(s), \quad \text {and} \quad {\mathfrak {m}}_2 = \underset{{s\ge 1}}{\arg \max } f(s). \end{aligned}$$

These quantities are then utilized to train a classifier for biological signals such as EEG measurements. Thus, for each subject (instance), the total number of extracted features would be $5\times M \times J=5\times 3\times 19=285$ in our case.

At the final stage and to classify the subjects into binary classes, we propose an algorithm based on bootstrap-aggregated decision trees (BADT) where each tree has a structure as depicted in the following. To be more precise, in addition to “H” and “MDD” labels, we assume there exist a latent label called “Mixed”, i.e., a sub-type, whose consideration will significantly improve the performance and interpretability of the classification algorithm. Furthermore, two other classifiers based on Gaussian naive Bayes (GNB) and convolutionary neural networks (an Encoder-Decoder with FCN-8 architecture) were developed and tested alongside the BADT, whose performance are further reported in Table 3. 
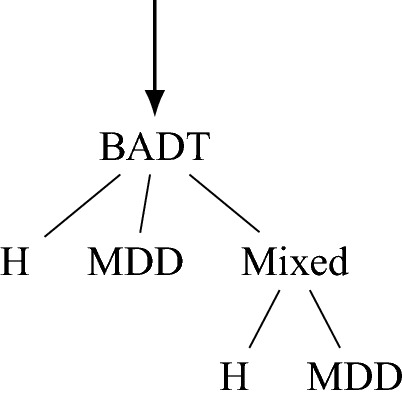

**Table 3:** Comparisons with different incorporated classifiers.

Classifier	Accuracy	Sensitivity	Specificity
Lehmer Transform + GNB	$93.8 \% \ (\pm 1.3)$	$93.9 \% \ (\pm 1.3)$	$93.8 \% \ (\pm 1.3)$
Lehmer Transform + CNN	$87.9 \% \ (\pm 1.5)$	${84.2}\% \ (\pm 1.9)$	$97.2 \% \ (\pm 1.3)$
Lehmer Transform + BADT	$90.5 \% \ (\pm 1.3)$	${94.1}\% \ (\pm 1.2)$	$87.0 \% \ (\pm 1.5)$

Besides, the last row of the Table 4 reports computational results of the proposed algorithm for classification of the benchmark instances using 10-fold cross validation averaged over 100 replications. Table 4 further compares the performance of our proposed framework to other methods from the literature, including the prominent work of Mumtaz et al.^17^. It is noted that all the reported results pertain to the experiments conducted on the same benchmark EEG dataset and under identical settings 10-fold cross validation with 100 replications.

On the basis of Table 4, it is seen that our proposed method achieves higher accuracy as compared to the method based on the wavelet transforms as well as other previously-proposed approaches for classifying the MDD and healthy subjects. A distinguishing aspect of our proposed Lehmer-based classifier is that it yields relatively lower standard errors when classifying the instances. In turn, this makes the results obtained by using the proposed classifier reliable to a great extent, and such reliability can potentially lead to an effective applications of the Lehmer-based classifiers by practitioners who monitor the possible impacts of prescribed antidepressants’ treatment on their patients.

By further noting that the classification algorithm proposed by Mumtaz et al., is mainly based on a logistic regression algorithm wherein the authors have used a combination of wavelet transforms (WT), short-time Fourier transforms (STFT) and empirical mode decomposition (EMD) as the feature extraction method and a rank-based algorithm for the purpose of feature selection, the superiority of Lehmer transform and its applications become more evident as compared to the frequency-based approaches.

**Table 4:** Comparisons with other algorithms from literature.

Derived EEG measure	Accuracy	Sensitivity	Specificity
ATR Index^13^	$61.7 \% \ (\pm 9.7)$	${70.0}\% \ (\pm 15.3)$	$54.0 \% \ (\pm 17.2)$
EEG Theta Coherence^16^	$70.7 \% \ (\pm 6.5)$	${75.7}\% \ (\pm 9.1)$	$65.7 \% \ (\pm 8.3)$
Coherence, PSD, PSD ratio^14^	$72.1 \% \ (\pm 7.6)$	${80.0}\% \ (\pm 13.5)$	$65.0 \% \ (\pm 12.3)$
P300 (amplitude and latencies)^2^	$74.2 \% \ (\pm 13.1)$	${70.0}\% \ (\pm 15.6)$	$75.0 \% \ (\pm 7.7)$
PSD, PSD ratios^14^	$54.5 \% \ (\pm 8.0)$	${55.0}\% \ (\pm 14.7)$	$50.0 \% \ (\pm 15.6)$
Wavelet Transform^17^	$87.5 \% \ (\pm 7.1)$	$95.0\% \ (\pm 4.3)$	$80.0 \% \ (\pm 8.8)$
Lehmer Transform	$\mathbf {93.8} \% \ (\pm \mathbf {1.3})$	$93.9 \% \ (\pm \mathbf {1.3})$	$\mathbf {93.8} \% \ (\pm \mathbf {1.3})$

Significant values are in bold.

## Conclusion

We propose a new transform that is shown be suitable to handle highly oscillatory non-stationary time series. It can effectively summarize and extract features from multi-channel signals and form the basis for building a highly accurate classifier. Although our work is still exploratory in nature, we believe that the potential of this new transform can be utilized fully in order to discern subtle patterns that cannot be recognized by other traditional methods such as Fourier and Wavelets transforms.
